# Experimental Evolution of Multidrug Resistance in *Neurospora crassa* under Antifungal Azole Stress

**DOI:** 10.3390/jof8020198

**Published:** 2022-02-18

**Authors:** Mi Zhou, Chengcheng Hu, Yajing Yin, Jingji Wang, Shuting Ye, Yifa Yu, Xianyun Sun, Shaojie Li

**Affiliations:** 1 Institute of Microbiology, Chinese Academy of Sciences, Beijing 100101, China; zhoumi0513@163.com (M.Z.); hucc@im.ac.cn (C.H.); jinji1236@163.com (J.W.); yeshuting19@mails.ucas.ac.cn (S.Y.); 2 College of Life Sciences, University of Chinese Academy of Sciences, Beijing 100049, China; 3 Tianjin Key Laboratory of Food Biotechnology, College of Biotechnology and Food Science, Tianjin University of Commerce, Tianjin 300134, China; yinyj20210628@tjcu.edu.cn; 4 Nanning Harworld Biological Technology Co., Ltd., Nanning 530007, China; yuyf@harworld.com

**Keywords:** experimental evolution, azoles, multidrug resistance, *Neurospora crassa*

## Abstract

Multidrug resistance, defined as the resistance to multiple drugs in different categories, has been an increasing serious problem. Limited antifungal drugs and the rapid emergence of antifungal resistance prompt a thorough understanding of how the occurrence of multidrug resistance develops and which mechanisms are involved. In this study, experimental evolution was performed under single-azole-drug stress with the model filamentous fungus *Neurospora crassa*. By about 30 weeks of continuous growth on agar plates containing ketoconazole or voriconazole with weekly transfer, four evolved multidrug-resistant strains 30thK1, 30thK2, 26thV1, and 24thV2 were obtained. Compared to the ancestral strain, all four strains increased resistance not only to commonly used azoles, including ketoconazole, voriconazole, itraconazole, fluconazole, and triadimefon, but also to antifungal drugs in other categories, including terbinafine (allylamine), amorolfine (morpholine), amphotericin B (polyene), polyoxin B (chitin synthesis inhibitor), and carbendazim (β-tubulin inhibitor). After 8 weeks of growth on agar plates without antifungal drugs with weekly transfer, these evolved strains still displayed multidrug-resistant phenotype, suggesting the multidrug resistance could be stably inherited. Transcriptional measurement of drug target genes and drug transporter genes and deletion analysis of the efflux pump gene *cdr4* in the evolved strains suggest that overexpression of *cdr4* played a major role in the resistance mechanisms for azoles and terbinafine in the evolved strains, particularly for 30thK2 and 26thV1, and evolved drug-resistant strains had less intracellular ketoconazole accumulation and less disruption of ergosterol accumulations under ketoconazole stress compared to wild type. Mutations specifically present in evolved drug-resistant strains were identified by genome re-sequencing, and drug susceptibility test of knockout mutants for most of mutated genes suggests that mutations in 16 genes, functionally novel in drug resistance, potentially contribute to multidrug resistance in evolved strains.

## 1. Introduction

Diseases caused by fungal pathogens seriously threaten human health, especially in the immune deficiency patients, and kills over 1.5 million people per year [1]. Fungal pathogens destroy ~20% agricultural yield worldwide, with a further 10% loss during postharvest, and the contamination of mycotoxigenic fungi seriously threatens food security [2,3,4]. Recent studies have proposed that pathogenic fungi cause a decline of species diversity [5,6]. More than 501 amphibian species declined over the past half-century, including 90 presumed extinctions [5], which is caused by chytridiomycosis panzootic, a lethal fungal disease. Diseases caused by fungal pathogens have drawn increasing concern in clinic, agriculture, and ecological environments.

In the clinic environment, five classes of antifungal drugs: azoles, allylamines, morpholines, polyenes, and echinocandins, are commonly used. Azoles, composed of both imidazoles (e.g., ketoconazole) and triazoles (e.g., voriconazole), are the most widely used drugs. Azoles inhibit ergosterol biosynthesis by targeting lanosterol 14-α-demethylase (ERG11) [7]. Allylamines (e.g., terbinafine) and morpholines (e.g., amorolfine) are also inhibitors of ergosterol biosynthesis. Allylamines target squalene epoxidase (ERG1), while morpholines inhibit both Δ^7^–Δ^8^ isomerase (ERG2) and Δ^14^-reductase (ERG24) [8]. Polyenes (e.g., amphotericin B) strongly bind to ergosterol, and thus create drug–lipid complexes to form a membrane spanning channel on the fungal cell membrane [7]. Echinocandins (e.g., caspofungin) disrupt cell wall biosynthesis by inhibiting (1, 3)-β-D-glucan synthase [7]. In addition, other antifungals are also used. Polyoxins, chitin synthesis inhibitors, which are produced by *Streptomyces cacaoi var. Asoeinsis* [9], have been widely used in the field for Botrytis disease, Brown Spot, and other fungal diseases as environmental friendliness [10,11]. Carbendazim, a β-tubulin inhibitor, was once extensively applied in the field because of its broad spectrum of antifungal activity [12,13].

Treatment by antifungals promotes the occurrence of drug resistance [1,14]. The criteria of drug resistance vary depending on purposes. In the clinic environment, epidemiological cutoff values (ECOFFs) or breakpoints are used as criteria for therapeutic guidance, while in research laboratories studying the mechanisms of antifungals, drug resistance refers to a strain that is less susceptible to a drug than a control or a reference strain [15]. The introduction of additional antifungal drugs strongly drives the development of multidrug resistance in the clinical and laboratory isolates [16,17]. For example, during voriconazole treatment, a drug-resistant *Candida glabrata* strain NRZ-2016–057 was isolated from a patient. It showed resistance to azoles, including posaconazole, voriconazole, itraconazole, and fluconazole. After the patient was switched from azole to echinocandin therapy, a new drug-resistant strain NRZ-2016–058 was isolated, displaying multidrug resistance to both azoles and echinocandins [16]. The reports of multidrug resistance to azoles, echinocandins, and polyenes are increasing, especially in the most prevalent fungal pathogens, *Candida* genus [17,18]. Since 2009, the global outbreak of “superbug fungus” infection caused by *C. auris*, which possesses characteristics of multidrug resistance and high mortality, has caused consternation in the medical community [17,19]. *Aspergillus fumigatus* forced by agricultural fungicides in the environment also obtained resistance to medical azoles [20]. Among *Aspergillus* species, there have been a few cases about multidrug resistance to azole and amphotericin B, or amphotericin B and caspofungin [17,21]. However, the mechanisms of multidrug resistance require extensive exploration.

During a process of adaptive evolution to the treated drugs, acquired drug resistance in fungi is a step-wise adaptation from physiological changes to genetic mutations [22]. For a better understanding of the fungal response to drugs and genetic researches on the mechanisms, scientists inclined to the view that the tolerance was unstably inherited adaptation, such as physiological changes including biochemical homeostasis and expression noise, and epigenetic mechanism, while the resistance was stably inherited adaptation [15,23]. Experimental evolution is a convenient and feasible approach to monitor the evolution of drug resistance [7,24]. Through this approach, different trajectories from the initially identical isolate were depicted and the emergence of resistance was discussed [25,26]. Moreover, the novel genes related to drug resistance were found by this approach [25,27].

*Neurospora crassa* has served as a model filamentous fungus in genetics, developmental biology, and molecular biology due to its advantages of easy transformation, easy sexual mating, and public availability of knockout mutants for more than two third of genes [28]. Using *N. crassa* as a model, it brings great convenience for screening drug target genes and functional analysis of drug stress responsive genes. The novel transcription factor ADS-1, positively regulating the transcriptional responses of azole efflux pumps and targeting genes both in *Aspergillus flavus* and *Fusarium verlicillioides*, was found and characterized for the first time in *N. crassa* [29]. Moreover, the functions of *ads-4*, *csp-1 ccg8,* and *stk-17*, which regulate the adaptive responses to antifungal azoles, were found in *N. crassa* and were demonstrated to be functionally conserved in pathogenic fungi [30,31,32,33]. Thus, *N. crassa* has advantages in the study mechanisms of antifungal resistance, particularly in filamentous molds. In this study, the experimental evolution in artificial populations for monitoring the evolution of multidrug resistance in real time was performed in *N. crassa*. Four evolutionary lineages were obtained with genetic stable multidrug resistance to not only antifungal azoles but also antifungals in other different categories, including terbinafine, amorolfine, amphotericin B, polyoxin B, and carbendazim. To explore the causes to drug resistance in the evolved strains, mutations were revealed through genome re-sequencing, the resistance-related mutations were identified by testing the drug susceptibility of knockout strains for the mutated genes, and transcript levels of drug target genes and drug transporter genes were analyzed.

## 2. Materials and Methods

### 2.1. Strains and Cultural Conditions

*N. crassa* strains used in this study and the related information are listed in Table 1. The wild-type (WT) strain FGSC#4200 and the deletion mutant of *cdr4* (*cdr4*^KO^, FGSC#11238) were obtained from Fungal Genetic Stock Center (FGSC). The overexpression strain of *cdr4* was constructed using the plasmid pCB1532-Pcfp-*cdr4*-TrpC [32]. Briefly, the plasmid was transformed into the WT strain by electroporation [34]. The *cdr4* deletion strains were constructed using knockout fragment *cdr4-Hph*, amplified with *cdr4*^KO^-5F/*cdr4*^KO^-3R from a fusion fragment that was fused by three fragments, including *cdr4* upstream and downstream flanking regions (amplified by *cdr4*^KO^-5F/*cdr4*^KO^-5R and *cdr4*^KO^-3F/*cdr4*^KO^-3R, respectively), and hygromycin encoding region (amplified by *cdr4*^KO^-HphF/*cdr4*^KO^-HphR) (Table S1). All the evolved strains: 30thK1, 30thK2, 26thV1, 24thV2, 30thC1, and 30thC2 were screened from a single isolate of the WT strain in this study (Table 1). The strains were cultured on Vogel’s medium (Vogel’s minimal salts supplemented with 2% [m/v] sucrose for slants or glucose for plates) solidified with 1.5% agar [35]. Liquid Vogel’s medium (Vogel’s minimal salts supplemented with 2% [m/v] glucose) was used for culturing mycelia of the strains. Sorbose plates (Vogel’s minimum medium, supplemented with 20 g/L sorbose, 0.5 g/L fructose, and 0.5 g/L glucose) were used for transformant growth after electroporation [34]. All cultures were incubated at 28 °C.

### 2.2. Experimental Evolution of Azole Resistance

Six independent populations of *N. crassa* were developed from a single azole-susceptible isolate of the WT strain. Conidia (1.0 × 10^6^–10^7^) of the WT strain were inoculated on Vogel’s medium at 28 °C. The lineage K1 and K2 were evolved by culturing the WT strain on plates with a stepwise increased concentration of ketoconazole (KTC, Sigma, Merck KGaA, Darmstadt, Germany); the lineages V1 and V2 were evolved by culturing the WT strain on plates with a stepwise increased concentration of voriconazole (VRC, Sigma); the lineage C1 and C2 were evolved by culturing the WT strain on plates with no drug. The experimental evolutionary populations were propagated for nearly 30 transfers (approximately 1 week incubation before each transfer).

### 2.3. Drug Susceptibility Test

KTC, VRC, fluconazole (FLC, Sigma), itraconazole (ITC, Merck millipere, Darmstadt, Germany), amorolfine (AMO, TCI, Shanghai, China), amphotericin B (AmB, Sigma), and caspofungin (CAS, BioVision, San Francisco, USA), were dissolved in dimethyl sulphoxide (DMSO). Terbinafine (Terb, J&K Scientific, Beijing, China) was dissolved in methanol. Polyoxin B (PoxB, Baoli’an, Tianjing, China) was dissolved in ddH_2_O. Triazolone was dissolved in isopropanol. Carbendazim (MBC, Sigma) was dissolved in 0.01 M HCl. These drug solutions were then sterilized by filtration. The drug solutions were then added to Vogel’s medium to make agar plates with different drugs. The final solvent concentration in media was below 0.25% (*v/v*). Two microliters of conidial suspension (2 × 10^6^ conidia/mL) collected from approximately 7-day-old cultures on slants were inoculated on the center of plates with or without the above drugs, and plates were incubated at 28 °C in the dark. Each experiment was independently repeated three times. The images and diameters of colonies were documented after a certain time of incubation.

$$\mathrm{Relative}\;\mathrm{inhibitory}\;\mathrm{rate}=(1-\frac{\mathrm{diameter}\;\mathrm{of}\;\mathrm{colony}\;\mathrm{in}\;\mathrm{plate}\;\mathrm{with}\;\mathrm{drug}}{\mathrm{growth}\;\mathrm{time}}÷\frac{\mathrm{diameter}\;\mathrm{of}\;\mathrm{colony}\;\mathrm{in}\;\mathrm{control}\;\mathrm{plates}}{\mathrm{growth}\;\mathrm{time}})\;\times \;100\%.$$

The minimum inhibitory concentration (MIC) values of drugs were determined in 96-well plates referring to Clinical and Laboratory Standards Institute (CLSI) broth microdilution method (M38-A2 document) with some modifications. The liquid Vogel’s medium was used to dilute the drugs and spores at designated concentrations. The 96-well plates were incubated at 28 °C for 40 h and followed by microscopic observation.

### 2.4. RNA Extraction and Quantitative Real-time RT-PCR (qRT-PCR)

Samples for RNA extraction were prepared as previously described with some modifications [33]. First, conidia were inoculated in the 9 cm petri dish containing liquid Vogel’s medium. After 24 h of stationary incubation at 28 °C in the dark, a mycelial mat on the surface of the medium formed and was then cut into circular pieces (Φ2-mm). About 15 pieces were transferred to 100 mL fresh Vogel’s medium and incubated at 28 °C with shaking at 200 rpm for 24 h. Mycelium was then harvested and freshly frozen in liquid nitrogen for RNA extraction. To assess transcriptional changes under drugs treatment, the samples were added with the antifungal drug (KTC or PoxB) after 12 h of incubation, and after another 12 h, mycelium was harvested for RNA extraction. Total RNA was extracted according to the standard TRIzol protocol (Invitrogen, Carlsbad, CA, USA). The cDNAs were synthesized from total RNA (2 μg) using a cDNA synthesis kit (FastQuant RT Kit (with gDNase), TIANGEN, Beijing, China) following the manufacturer’s protocol. The qRT-PCR analysis was performed on a CFX96 multicolor real-time PCR detection system (Bio-Rad, Hercules, CA, USA) with SYBR green detection (KAPA SYBR^®^ FAST qPCR Kits; KAPA BIOSYSTEMS, Boston, MA, USA) according to the manufacturer’s instructions. Gene-specific primers were designed using the online tools PrimerQuest or Primer 5 and are listed in Table S2.

### 2.5. HPLC-MS Analysis of Sterol Contents and Ketoconazole Accumulation

Samples for sterol extraction were prepared as that for RNA extraction described above. Mycelial samples were heat-dried at 80 °C and then ground into fine powder using a mortar and pestle. About 10 mg of mycelial powder was used for sterol extraction in Agilent vials (2 mL). Extraction and chemical analysis of sterols and KTC were performed as previously described [36] using ergosterol (J&K Scientific, Beijing, China) and KTC standards. The statistical analysis was conducted as previously described [36].

### 2.6. The Next-Generation Whole-Genome Resequencing (NGS)

Genomic DNA was extracted from mycelia grown in liquid Vogel’s medium by phenol/chloroform extraction technique [37]. One microgram of genomic DNA was randomly fragmented by Covaris. Genomic DNA fragments with an average size of 200–400 bp were selected with magnetic beads. DNA was quantified by Qubit fluorometer. Fragments were end repaired and then 3′ adenylated. Adaptors were ligated to the ends of the 3′ adenylated fragments. Fragments with adaptors were amplified with PCR, and the PCR products were purified by magnetic beads. The double-stranded PCR products were heat denatured and circularized by the splint oligo sequence. The single strand circle DNA (ssCir DNA) was formatted as the final library, which was then qualified by quality control. DNA sequencing was performed on DNBSEQ platform (BGI, Shenzhen, China). After filtering low quality reads, each dataset with a coverage depth of over 50×, a high mapping rate (97.69–98.38%), and a high unique rate (over 99.51%) were aligned to the *N. crassa* reference genome NcrassaOR74A to detect SNPs and Indels.

## 3. Results

### 3.1. N. crassa Acquired Multidrug Resistance under Azole Stress

**Figure 1:**
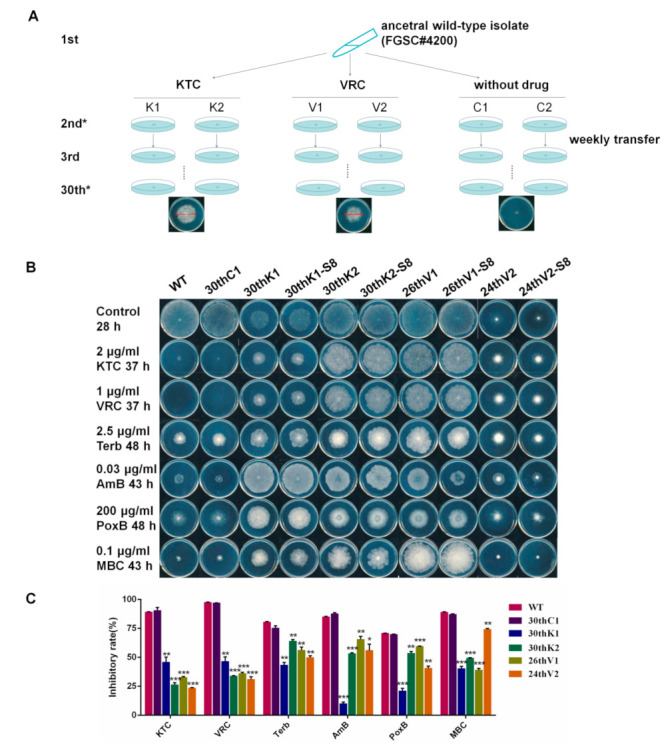
Acquired multidrug resistance in *N. crassa* under azole stress. (**A**) Flow chart for the development of azole-resistant strains. (**B**) Drug susceptibility test of the indicated strains to different antifungals at designated concentrations. Two microliter aliquots of conidial suspension (2 × 10^6^ conidia/mL) were inoculated in the center of plates (ϕ90-mm) with or without the antifungals. The plates were then incubated at 28 °C for the indicated time. The experiment was independently repeated at least three times. (**C**) Relative growth inhibition rates were calculated based on colony diameters of the strains grown on plates with and without antifungals at indicated growth time. Values from three replicates were used for statistical analysis. Means of the inhibition rates are shown, and standard deviations are marked with error bars. Difference significance between the evolved strains and the ancestral WT strain were estimated by the *t*-test and marked as * (*p* < 0.05), ** (*p* < 0.01), and *** (*p* < 0.001). The abbreviation the antifungal drugs are explained: KTC (ketoconazole), VRC (voriconazole), Terb (terbinafine), AmB (amphotericin B), PoxB (polyoxin B), and MBC (carbendazim).

To rapidly obtain the experimental evolutionary strains, the ancestral WT strain was cultured on Vogel’s agar plates with KTC (labeled with “K”) or VRC (labeled with “V”), while the same strain grown on Vogel’s agar plates without any antifungal drug was used as the control (labeled with “C”). Two biological replicates were applied for each treatment (labeled with “1” or “2”). The strains were weekly transferred nearly 30 times, during which the concentration of KTC and VRC in the agar plates was continuously increased (the evolutionary process was displayed in Figure 1A). Four evolved strains with increased azole resistance, including 30thK1, 30thK2, 26thV1, and 24thV2, were obtained. Drug susceptibility test showed that the MIC values of WT for KTC and VRC were 3.6 μg/mL and 1.4 μg/mL, respectively (Table 1); while the MIC values for KTC were respectively 9.0, 10.8, 11.8, and 20 μg/mL in 30thK1, 30thK2, 26thV1, and 24thV2, which had at least 2.5-fold increase over that in the ancestral WT strain; and the MIC values for VRC were respectively 4.8, 4.6, 6.4, and 9.5 μg/mL in 30thK1, 30thK2, 26thV1, and 24thV2, raised by 3.4–7-fold over that in WT. In addition to KTC/VRC resistance (Figure 1B), the evolved strains also showed increased resistance to other azoles including itraconazole (ITC), fluconazole (FLC), and triadimefon (TDF) compared to WT (Figure S1). In contrast, after 30 weeks of growth on azole-absent agar plates, the evolved control strains 30thC1 and 30thC2 still had the same MIC values for KTC and VRC as the ancestral WT strain (Table 1). Since 30thC1 and 30thC2 did not show any phenotypic difference, only 30thC1 was used for further study. On agar media without azoles, 30thK1 and 24thV2 grew dramatically slower than WT, while 30thK2 and 26thV1 grew almost as normally as WT (Figure 1B). However, on agar plates with KTC or VRC, the four evolved strains displayed faster growth than WT and 30thC1, and their inhibition rates by these antifungal azoles were significantly lower than those of WT and 30thC1 (Figure 1C).

We also tested the sensitivities of these evolved strains to antifungals in other categories. Interestingly, when grown on the agar plates, the four evolved strains also showed increased resistance to terbinafine (Terb), amorolfine (AMO), amphotericin B (AmB), polyoxin B (PoxB), and carbendazim (MBC), as compared to 30thC1 and WT (Figure 1B). Notably, the 30thK1 strain showed the most significant increase in resistance to Terb, AmB, PoxB, and AMO. The inhibition rates of 30thK1 by these antifungal drugs were respectively 43.39%, 10.00%, 21.05%, and 26.58%, while those of WT were 80.46%, 85.05%, 70.84%, and 74.02%, respectively (Figure 1, Figure S1). However, the sensitivity of these evolved strains to caspofungin (CAS) was not different from 30thC1 and WT (Figure S1).

### 3.2. The Multidrug Resistance Developed from Experimental Evolution Is Stably Inherited

In order to test whether the acquired drug resistance is stably inherited, the four evolved strains were cultured on antifungal-absent Vogel’s agar plates with weekly transfer. As shown in Figure S1C, after 8 weeks of growth on the medium (S8), the sensitivities of 30thK1, 30thK2, and 26thV1 to all above mentioned drugs were not changed, indicating that their multidrug resistance was stably inherited. However, the sensitivity of the strain 24thV2-S8 to KTC was obviously increased compared to its initial strain 24thV2. The growth inhibition rate increased from 23.45% to 32.93% in the 24thV2-S8 strain relative to 24thV2, but it was still significantly lower than that in the WT strain (Figure 1B and Figure S1C). The sensitivities of 24thV2-S8 to the other antifungal drugs mentioned above were not changed relative to 24thV2.

### 3.3. Developmental Processes of Multidrug Resistance in N. crassa Stressed by Different Antifungal Azoles

**Figure 2:**
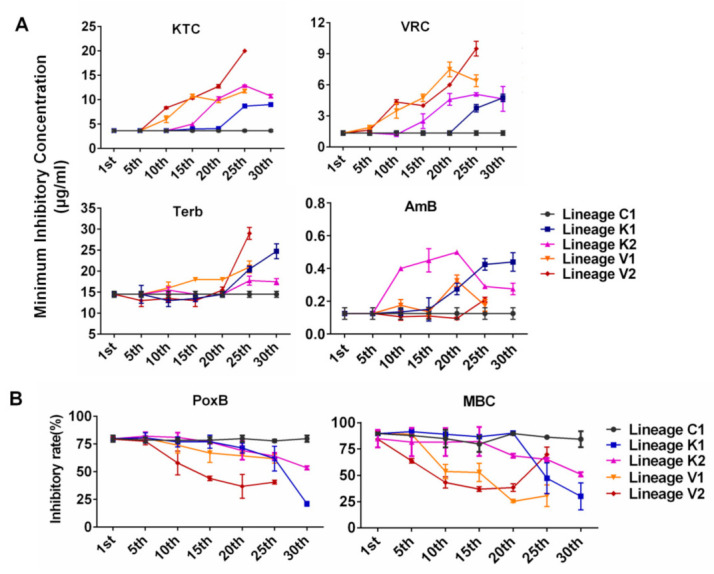
The developmental processes of resistance to different antifungal drugs. (**A**) The minimum inhibitory concentrations of different antifungal drugs, including KTC, VRC, Terb, and AmB, were measured once every 5 weeks in evolved strains exposed to KTC (Lineage K1/2) or VRC (Lineage V1/2). (**B**) Relative growth inhibition rates of evolved strains were tested for PoxB and MBC once every 5 weeks. The lineages K1/2 were respectively marked with blue square and purple triangle, and the lineages V1/2 were respectively marked with orange triangle and brown diamond. The experiment was independently repeated at least three times.

In order to track the developmental processes of multidrug resistance in the evolved strains, the susceptibilities of four evolutionary lineages K1, K2, V1, and V2 to KTC, VRC, Terb, AmB, PoxB, and MBC were tested once every 5 weeks during experimental evolution. For KTC, VRC, Terb, and AmB, the MIC values were tested in 96-well plates by observing conidial germination (Figure 2A). For PoxB and MBC, the growth inhibition was observed on agar plates with these drugs, and the inhibitory rates were calculated based on colony size (Figure 2B), since conidial germination is not easily observed in the liquid medium containing PoxB or MBC. As shown in Figure 2, the evolutionary lineages K1, K2, V1, and V2 followed different evolutionary trajectories and reached different levels of resistance to each antifungal drug. Three regularities of drug-resistance occurrence in the laboratory evolutionary strains are summarized below:

1. Evolutionary direction and trajectory were random. As shown in Figure 2, for each of the tested drugs, evolutionary curves in the four lineages are different and the increased resistance to each drug in four lineages initially appeared at different generations. For example, lineages K1 and K2 evolved from the same strain under KTC stress and developed different levels of resistance for each drug (KTC, VRC, Terb, AmB, PoxB, and MBC). The lineage K1 started to exhibit increased resistance to KTC and VRC at the 25th generation, while the lineage K2 initially showed increased resistance to these azoles at the 15th generation (Figure 2). The lineage K1 rapidly increased Terb resistance after the 20th generation, while Terb resistance in K2 was slightly increased after the 20th generation. For AmB resistance development, the lineage K2 sharply increased AmB resistance after the 5th generation, while AmB resistance in K1 slowly increased from the 5th generation and then rapidly increased after the 15th generation. Both K1 and K2 started to exhibit PoxB resistance at the 20th generation. PoxB resistance in K1 rapidly raised after the 25th generation and reached a peak at the 30th generation, while K2 stably increased PoxB resistance till the 30th generation. MBC resistance in the lineage K1 sharply increased after the 20th generation, while K2 slowly increased MBC resistance from the 15th generation.
2. The development of cross resistance to the azoles frequently occurred in *N. crassa*. In each evolutionary lineage, KTC resistance and VRC resistance almost concurred and increased with the similar developing curve. For example, in the lineage K1, the resistance to both KTC and VRC started to be observed after the 20th generation and quickly increased from the 20th to 25th generation (Figure 2A). A similar case also happened to the evolutionary lineages K2, V1, and V2, although the generation of resistance occurrence varied from one lineage to another.
3. The resistances to PoxB and MBC were concurrent to azole resistance. As shown in Figure 2B, the lineage K1 moderately increased resistance to PoxB at the 20th generation, in which KTC resistance in this lineage started to be detected. After the 20th generation, MBC resistance also appeared. In the lineage K2, PoxB resistance and KTC resistance were concurrent after the 15th generation, while MBC resistance occurred after the 20th generation. The resistance to PoxB, MBC, and KTC simultaneously appeared in V1 and V2.

### 3.4. Transcriptional Changes of Drug Target Genes in the Evolved Strains

To understand the mechanisms of multidrug resistance to the azole, AmB, Terb, MBC, and PoxB in the evolved strains, we firstly analyzed the changes in the drug target genes, including *erg11* (azoles), *erg1* (Terb), *β-tubulin* (MBC), and chitin synthesis (*chs*) genes (PoxB). Through fragment sequencing, no mutation in these genes was found in these evolved strains (data not shown).

**Figure 3:**
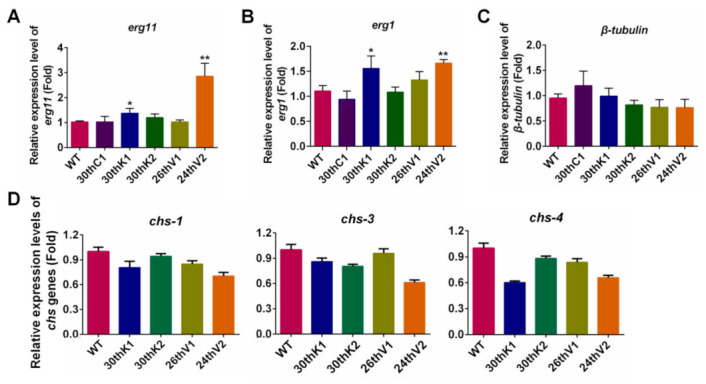
The transcript levels of drug target genes in the evolved strains. Transcript levels of (**A**) *erg11*, (**B**) *erg1*, (**C**) *β-tubulin*, and (**D**) chitin synthesis (*chs*) genes, including *chs-1*, *chs-3*, and *chs-4*, were measured by qRT-PCR, calculated by 2^-^^ΔΔCt^ method and normalized to *β-tubulin*. The gene *β-tubulin* was normalized to *vma-1*. The results presented here are means of three biological replicates. The significant levels were calculated by t-test and marked as * (*p* < 0.05), ** (*p* < 0.01).

Then, transcriptional levels of these target genes were measured by qRT-PCR. For the azole target gene *erg11*, its transcript levels in 30thK1 and 24thV2 were significantly higher than that of WT (with a 1.4-fold and 2.8-fold increase, respectively), while transcript levels of *erg11* in 30thK2 and 26thV1 had no significant difference from those of WT and 30thC1 (Figure 3A). For the Terb target gene *erg1*, the transcript levels in 30thK1 and 24thV2 were 1.6 and 1.7-fold higher than that in WT, while transcript levels of *erg1* in the 30thK2 and 26thV1 had no significant difference from those of WT and 30thC1 (Figure 3B). Thus, the resistance to azoles and Terb in 30thK1 and 24thV2 might be associated with raised expression of *erg11* and *erg1*, while the azole and Terb resistance in the 30thK2 and 26thV1 strains might be attributed to mechanisms unrelated to the above drug target genes.

Transcript levels of *β-tubulin* (the target gene of MBC) and chitin synthetic genes *chs-1*, *chs-3,* and *chs-4* (the target genes of PoxB) in the four evolved strains were similar to those in WT (Figure 3C,D). Thus, the acquired resistance to MBC and PoxB in these evolved strains was not caused by transcriptional changes in their target genes.

### 3.5. Accumulation of Ergosterol and Sterol Intermediates in the Evolved Strains

**Figure 4:**
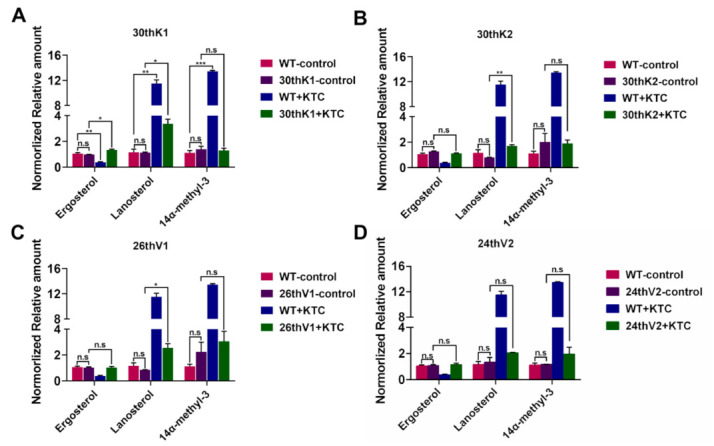
Accumulation of ergosterol, lanosterol and 14α-methyl-3,6-diol (14α-methyl-3) in the evolved strains (**A**) 30thK1, (**B**) 30thK2, (**C**) 26thV1, and (**D**) 24thV2, and in the ancestral WT strain under KTC stress. Sterols were measured by HPLC-MS. The results presented here are means of two biological replicates. The significant levels were calculated by *t*-test and marked as * (*p* < 0.05), ** (*p* < 0.01), and *** (*p* < 0.001).

The contents of ergosterol and the intermediates of ergosterol biosynthesis are commonly associated with resistance to inhibitors of ergosterol biosynthesis and polyenes [38]. To analyze the changes in cellular accumulation of ergosterol and the intermediates in the azole-evolved strains, sterols were measured by HPLC-MS. When grown in Vogel’s liquid medium without any antifungal drug, the total ergosterol, lanosterol, and 14α-methyl-3 (14α-methyl-3,6-diol) amounts in the four evolved strains (30thK1, 30thK2, 26thV1, and 24thV2) maintained similar levels to those in WT (Figure 4). After 12 h of 2 μg/mL KTC treatment in liquid Vogel’s medium, the cellular ergosterol content in WT was reduced to only 37.53% of that in the no-drug control, but lanosterol and 14α-methyl-3 contents were increased by 9.89 and 12.03 folds, respectively (Figure 4A). In contrast, KTC treatment did not cause a significant change of ergosterol contents in 30thK2, 26thV1, and 24thV2. Ergosterol in 30thK1 displayed a 1.36-fold increase after KTC treatment (Figure 4B–D). Although lanosterol levels respectively increased by 2.98, 2.13, and 2.98 folds in 30thK1, 30thK2, and 26thV1 after KTC treatment, the increased levels were largely lower than that of WT. Lanosterol level of 24thV2 was not changed by KTC treatment. For 14α-methyl-3, no significant change was detected in the four evolved strains after KTC treatment (Figure 4). These results indicate that sterol composition in evolved drug-resistant strains was less affected by KTC stress. Thus, they can maintain stable ergosterol synthesis under azole stress.

### 3.6. Transcriptional Changes of Transporter Genes for Drug Absorption and Efflux in Evolved Strains

**Figure 5:**
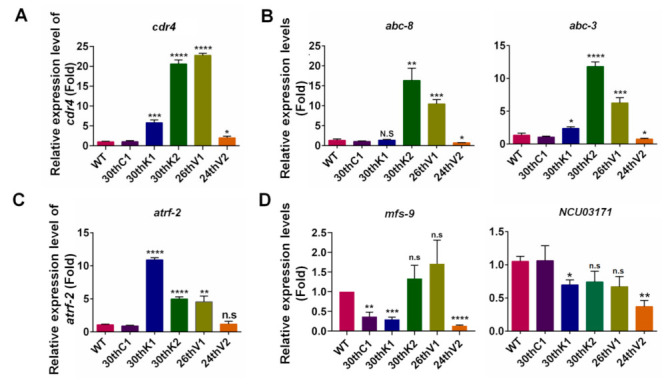
The transcript levels of the genes encoding transmembrane proteins. Transcript levels of (**A**) *cdr4*, (**B**) *abc-8* and *abc-3*, (**C**) *atrf-2,* and (**D**) *mfs-9* and *NCU03171* were measured by qRT-PCR, calculated by 2^−^^ΔΔCt^ method, and normalized to *β-tubulin*. The results presented here are means of at least three biological replicates. The significant levels were calculated by *t*-test and marked as * (*p* < 0.05), ** (*p* < 0.01), *** (*p* < 0.001), and **** (*p* < 0.0001).

Besides the drug targets, activation of drug efflux pumps is another prevalent cause to drug resistance in the clinical and experimental strains [1]. In *N. crassa*, ATP-binding cassette (ABC) transporter CDR4 was characterized as the major contributor to azole resistance [39]. Moreover, it was reported that the resistance to MBC in *Isaria fumosorosea* was caused by overexpression of *ifT1*, the homologous gene of *cdr4* [40]. To assess the role of *cdr4* in drug resistance in the evolved strains, transcript levels of *cdr4* were measured by qRT-PCR. As shown in Figure 5A, transcript levels of *cdr4* were significantly higher in all four evolved strains than that of WT. In 30thK2 and 26thV1 strains, *cdr4* displayed over 20-fold transcriptional increases relative to WT, and its transcript levels in 30thK1 and 24thV2 were increased by 5.6 and 2 folds relative to WT, respectively (Figure 5A).

*Microsporum canis* and *Trichophyton rubrum* strains with Terb resistance showed overexpression of genes encoding ABC transporters, including PDR1, MDR1, MDR2, and MDR4 [41,42]. Their homologue-encoding genes in *N. crassa* include *cdr4*, *abc-8,* and *abc-3*. To gain insight into the likely mechanisms to Terb resistance, transcript levels of *abc-8* and *abc-3* were analyzed by qRT-PCR. As shown in Figure 5B, *abc-8* transcript levels in 30thK2 and 26thV1 strains were respectively 12.3 and 7.9-fold of that in WT, and transcript levels of *abc-3* in 30thK1, 30thK2, and 26thV1 were respectively 1.8, 8.7, and 4.6 folds of that in WT. In contrast, transcript levels of these two genes were slightly down-regulated in 24thV2 compared to WT. To see the effects of deletion of these genes on drug sensitivity in *N. crassa*, drug susceptibility test was performed in three strains of gene knockout, including *cdr4*^KO^, *abc-8*^KO^, and *abc-3*^KO^. As shown in Figures S2A,B, the *cdr4*^KO^ strain was hypersensitive to KTC, VRC, and Terb, but had similar sensitivity to MBC as WT. The deletion mutants for *abc-8* and *abc-3* did not show significant difference from WT in the sensitivity to azoles and Terb (Figure S2A).

The transcript level of *atrf-2*, the homologous gene of *BMR1* in *Botrytis cinerea*, was also measured. It was reported that Δ*BMR1* mutant was hypersensitive to the antibiotic PoxB and the transporter encoded by *BMR1* may act as a PoxB efflux pump [43]. By qRT-PCR detection, transcript levels of *atrf-2* were increased by 10.0, 4.6, and 4.2-fold in 30thK1, 30thK2, and 26thV1 relative to WT, respectively (Figure 5C). The *atrf-2* knockout strain displayed increased sensitivity to PoxB, but had WT sensitivity to KTC or VRC (Figure S2C). PoxB is a peptidyl pyrimidine antibiotic that may be transported by peptide transporters [44], and peptide transporters on carriage of di-/tripeptide and oligopeptide and PoxB have been reported in *C. albcans* [45,46]. By alignment analysis of the homologous protein sequences, 13 genes encoding putative peptide transporters were identified in *N. crassa* (Table S3, Figure 5D). Among the available knockout mutants for 6 genes (*mfs-9*, *NCU03171*, *opt-3*, *opt-4*, *mfs-8,* and *NCU09874*), *mfs-9*^KO^ and *NCU03171*^KO^ showed obvious resistance to PoxB (Figure S2C). Transcript levels of *mfs-9* in 30thK1 and 24thV2 were decreased to 29.55% and 13.46% of that in WT. The gene *NCU03171* had lower transcript levels in the four evolved strains compared to WT. In the 30thK1 and 24thV2 strains, transcript levels of *NCU03171* were decreased by 69.99% and 37.00% respectively relative to that in WT (Figure 5D). Other peptide transporter encoding genes, including *mfs-8*, *NCU08397* (encoding the oligopeptide transporter [OPT] homologue), and *opt-1*, showed decreased transcript levels in all four evolved strains relative to those in WT. To be noted, transcript levels of *mfs-8*, *NCU08397,* and *opt-1* were reduced in response to PoxB treatment in WT (Table S3), suggesting that the transcriptional repression of these putative oligopeptide transporter genes might increase resistance to PoxB.

In summary, relative to WT, the great transcriptional changes of genes encoding transmembrane proteins, including drug efflux (*cdr4*, *abc-8*, *abc-3,* and *atrf-2*) and peptide transporters (such as *mfs-9* and *NCU03171*), occurred in the evolved strains.

### 3.7. Overexpression of cdr4 Plays a Vital Role in Azole and Terbinafine Resistance in the Evolved Strains

As transcriptional levels of *cdr4*, which encodes the major azole efflux pump, were significantly increased in the evolved strains, to explore the contribution of increased expression in *cdr4* to azole resistance in these strains, the *cdr4* overexpression strain (*cdr4*^OE^) was constructed. The drug susceptibility of *cdr4*^OE^, together with the *cdr4* knockout mutant (*cdr4*^KO^) and WT, was tested. As shown in Figure S3, in addition to being hypersensitive to KTC, *cdr4*^KO^ was also hypersensitive to Terb. In contrast, *cdr4*^OE^ displayed increased resistance to KTC and Terb compared to WT. However, both *cdr4*^KO^ and *cdr4*^OE^ exhibited similar susceptibility to MBC as WT, suggesting that CDR4 is important for efflux of both azoles and allylamine but not for other kinds of antifungals, like MBC.

**Figure 6:**
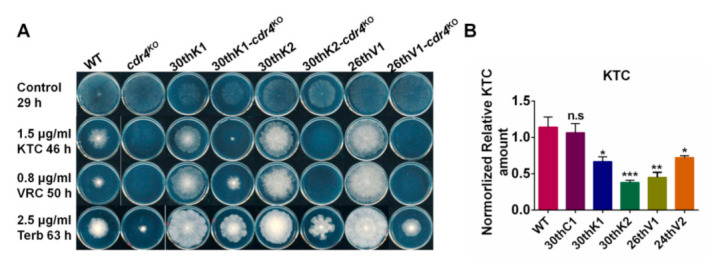
The contribution of *cdr4* to drug resistance in the evolved strains. (**A**) Drug susceptibility test in the indicated strains. Two microliter aliquots of conidial suspension (2 × 10^6^ conidia/mL) were inoculated in the center of plates (ϕ90-mm) with or without the antifungals. The plates were then incubated at 28 °C for the indicated time. The experiment was independently repeated at least three times. (**B**) Accumulations of KTC in the evolved strains and the ancestral WT strain under azole stress were measured by HPLC-MS. The results presented here are means of three biological replicates. The significant levels were calculated by t-test and marked as * (*p* < 0.05), ** (*p* < 0.01), *** (*p* < 0.001).

In order to test whether *cdr4* was involved in the development of drug resistance in these evolved strains, *cdr4* was deleted in the monokaryon strains of 30thK1, 30thK2, and 26thV1, respectively. We failed to obtain the monokaryon strain of 24thV2 due to its poor conidial production (Figure S1D). As shown in Figure 6A, deletion of *cdr4* in the strains 30thK1, 30thK2, and 26thV1 largely reduced the resistance to KTC, VRC, and Terb. Therefore, the increase of *cdr4* expression might play a vital role in azole and Terb resistance in the evolved strains.

Coinciding with the up-regulation of *cdr4* genes, the KTC accumulation in the four evolved strains was significantly lower than those in WT and 30thC1 under KTC stress. As shown in Figure 6B, after being cultured in the liquid medium with 2 μg/mL KTC for 12 h, the cellular KTC levels in the four evolved strains were only 62.27%, 41.37%, 37.36%, and 70.65% of that in WT, respectively.

### 3.8. Identification of Potential Resistance-Related Genes in Evolved Strains

In order to identify the other potential genes contributing to multidrug resistance, the genome of the resistant monokaryon isolates from 30thK1, 30thK2, and 26thV1 (monokaryon isolates were not obtained in 24thV2 due to poor conidial production), together with the control strain 30thC1 and the WT strain, were sequenced by NGS. More than 300 nonsynonymous and 60 Indels were detected in the evolved resistant strains and the control strain 30thC1, and 135 nonsynonymous and 64 Indel mutations were found in the ancestral WT compared to the standard strain sequences (http://fungidb.org/common/downloads/Current_Release/NcrassaOR74A/ accessed on 10 February 2021) (Table S4). By comparing the SNPs and the Indels in the resistant strains (30thK1, 30thK2 and 26thV1) and drug-sensitive strains (WT and 30thC1), the specific variations in each of the resistant strains were founded and listed in Tables S5 and S6.

In the 30thK1 strain, 1001 SNPs (673 synonymous and 328 nonsynonymous) and 69 Indels (27 inserts and 42 deletes) were found compared with the standard strain sequence. After eliminating the same SNPs and Indels in drug-sensitive strains (WT and 30thC1), 5 nonsynonymous and 28 Indels likely related to drug resistance were identified in this strain (Tables S5 and S6). Encoded proteins by mutated genes in 30thK1 include tRNA-processing-10 (*NCU02058*), DUF726 domain-containing protein (*NCU04410*), RIP defective protein (*NCU02034*), 3-hydroxyacyl-CoA dehydrogenase (*NCU16336*), DUF726 domain-containing protein (*NCU02065*), mitochondrial intermediate peptidase (*NCU02063*), zinc metallopeptidase (*NCU02060*), uridine nucleosidase Urh1 (*NCU02055*), transcription initiation factor (*NCU02052*), GTP-binding protein (*NCU02044*), sterol-4alpha-carboxylate 3-dehydrogenase (*NCU02042*), phosphoadenosine phosphosulfate reductase (*NCU02005*), ethanolaminephosphotransferase (*NCU01993*), SET-8 (*NCU01973*), tyrosine-protein phosphatase CDC14 (*NCU03246*), and 11 hypothetical proteins (Tables S5 and S6).

To investigate whether these genes are related to drug sensitivity, the drug susceptibilities of single gene deletion mutants to KTC, Terb, AmB, MBC, and PoxB were tested. Except for *NCU16336*, *NCU02063*, *NCU02044*, *NCU14007*, *NCU04410*, *NCU02014*, *NCU02005,* and *NCU07390*, whose mutants are either unavailable in FGSC or out of our mutant library, mutants for the rest genes were tested. As shown in Figure S4, Tables S5 and S6, mutants for 16 genes exhibited sensitivity different from WT to the tested drugs. The single gene deletion mutants for *NCU02055*, *NCU02012*, *NCU01993,* and *NCU03246* were only more resistant to KTC; the mutants for *NCU2052* and *NCU01947* were only more resistant to Terb; and the mutants for *NCU02058*, *NCU02051*, *NCU02060* and *NCU02026* were more resistant to both KTC and Terb, compared to WT. However, no mutant was more resistant to AmB, MBC, or PoxB than WT. The *NCU02012* deletion mutant was more sensitive to AmB, MBC, and PoxB than WT; and the *NCU02051* deletion mutant was more sensitive to PoxB than WT (Figure S4, Tables S5 and S6). These results suggest the drug resistance in the evolved strain 30thK1 is attributed to mutations at multiple genes.

In the 30thK2 strain, 977 SNPs (659 synonymous and 318 nonsynonymous) and 56 Indels (20 inserts and 36 deletes) were identified. After eliminating the same SNPs and Indels in WT and 30thC1, 6 nonsynonymous and 13 Indels likely related to drug resistance were isolated (Tables S5 and S6). To be noted, 30thK2 and 30thK1 shared 10 commonly mutated genes. These genes include *NCU02065*, *NCU02063*, *NCU02051*, *NCU02026*, *NCU02024*, *NCU02012,* and *NCU01993*, whose mutants were tested above, and three hypothetical protein encoding genes without available mutants (*NCU16667*, *NCU02014* and *NCU04216*) (Tables S5 and S6). The drug susceptibilities were tested for the available knockout mutants of eight mutated genes (*NCU08055*, *NCU01997*, *NCU09308*, *NCU02036*, *NCU02548*, *NCU03491*, *NCU03641* and *NCU02243*) in 30thK2. The mutants for *NCU03641* (beta-glucosidase 2), *NCU08055* (glycoside hydrolase family 3 protein ZIP-1), and *NCU02243* (hp) showed the WT sensitivity to all tested drugs; the mutants for *NCU01997* (ABC transporter) and *NCU09308* (glycoprotease) were more resistant to Terb than WT; and the mutants for *NCU02548* (hp) and *NCU03491* (RNA splicing factor) were more resistant to KTC than WT (Figure S4, Tables S5 and S6). In addition, the *NCU02548* deletion mutant was more sensitive to AmB, and the *NCU02036* (tRNA-splicing endonuclease) deletion mutant was more sensitive to both AmB and MBC, compared to WT.

In the 26thV1 strain, 993 SNPs (675 synonymous and 318 nonsynonymous) and 66 Indels (25 inserts and 41 deletes) were identified. After eliminating the same SNPs and Indels in WT and 30thC1, 7 nonsynonymous and 15 Indels likely related to drug resistance were isolated (Tables S5 and S6). The 26thV1 strain shared seven commonly mutated genes with 30thK1 (*NCU02034*, *NCU02065*, *NCU02055*, *NCU02033*, *NCU02014*, *NCU02005,* and *NCU14007*), and eight commonly mutated genes with the 30thK2 strain (*NCU02036*, *NCU02548*, *NCU03491*, *NCU16667*, *NCU03641*, *NCU02014*, *NCU02243,* and *NCU04216*). In addition, *NCU02051* and *NCU02024* had mutations in all three evolved strains. Four genes, including *NCU16302*, *NCU01967*, *NCU02867,* and *NCU05591* presented mutations only in 26thV1. *NCU16302* encodes the mitochondrial ribosomal protein S5 and its knockout mutant is unavailable in FGSC. NCU01967 encodes a hypothetical protein and its knockout mutant was more resistant to both KTC and PoxB but more sensitive to AmB than WT. *NCU02867* also encodes a hypothetical protein and its knockout mutant was more resistant to KTC, Terb, and AmB than WT. A nonsynonymous mutation in *NCU05591* (ABC transporter CDR4) was found in 26thV1, whose deletion mutant is hypersensitive to KTC and Terb (Figure S4).

## 4. Discussion

Drug resistance, which results in the failure of antifungal therapy, is a serious problem in the clinic and environment setting. The emergence of multidrug resistance makes this problem more serious and complicated. In the clinic setting, multidrug resistance has been generally considered as a consequence of applying different kinds of antifungals for the control of fungal infections [1,16]. However, in pathogenic yeast *C. parapsilosis*, cross-resistance to posaconazole, FLC, VRC, ITC, and echinocandin was developed under posaconazole stress by experimental evolution [47]. Thus, multidrug resistance in fungi can occur under stress of a single azole drug. This study also provides a noteworthy case with a different fungal species. Upon KTC or VRC stress, drug resistance in the model filamentous fungus *N. crassa* increased not only for other antifungal azoles and other ergosterol synthesis inhibitors with different action targets other than ERG11, such as Terb and AMO, but also for antifungals in other mechanisms, including AmB, PoxB, and MBC. Thus, the stress by a single azole drug can result in resistance acquisition to antifungal drugs in very broad categories. Fungal cells have different mechanisms to sense, respond, and adapt to the stresses from antifungal drugs with different targets. The fact that all the four strains evolved under single-azole-drug stress resulted in increased resistance to antifungal drugs in different categories compared to WT, suggests that the mechanisms used to protect fungal cells from antifungal drugs with different modes of action have crosstalk. In the complicated processes for drug sensing, responding, and defensing under the stresses from antifungal drugs in different categories, some common signaling components might participate, which might be the basis for the development of multidrug resistance. Some mutations that result in the resistance to antifungal drugs in one mode of action might also elevate the resistance to antifungal drugs in other mechanisms. Both the pathogenic yeast *C. parapsilosis* and the model filamentous fungus *N. crassa* can develop multidrug resistance within several weeks under continuous stress of azoles, which are the most widely used antifungals in clinic and in agriculture. Thus, the right way to reduce the risk of multidrug resistance formation should be taken into consideration.

Although drug ranges of resistance are similar in four *N. crassa* evolved strains, developing trajectories in different evolved strains varied. The evolved strains showed different levels of resistance, different gene expression pattern, and different SNPs and Indels, indicating that the evolution of drug resistance in different strains followed different trajectories. It is consistent with the previous conclusion that random processes in response to selective pressure determine the variation in the trajectory of drug resistance [26]. Random mutation or genetic modification of different adaptive strategies to azole stress might result in different trajectories of drug resistance in these evolved strains (Table 2).

For the emergence of azole and Terb resistance, the 30thK1 strain displayed slightly up-regulated expression of the target genes *erg11* and *erg1*, as well as the drug efflux pump encoding gene *cdr4*. The slight transcriptional up-regulation of the target genes and *cdr4* might have limited contribution to azole and Terb resistance. The *cdr4* deletion strain of 30thK1 still maintained moderate resistance to azoles. Other causes for the azole and Terb resistance might exist. The mutations in genes *NCU02058*, *NCU02060*, and *NCU02051* may be involved in both azole and Terb resistance in 30thK1 since their single gene deletion mutants showed resistance to both KTC and Terb (Figure S4). In addition, *NCU02012* (encoding a protein containing C6 zinc binuclear cluster DNA-binding domain) and *NCU02052* (encoding a transcription initiation factor), whose deletion mutants displayed increased resistance to KTC and Terb, respectively (Figure S4), are functionally related to transcriptional regulation, implying certain changes in transcriptional regulation may occur in 30thK1. The 30thK1 strain has a slower growth phenotype than WT on agar media without antifungals. It is possible that the growth defect is a fitness cost for drug resistance. Mutations correlative to resistance with a fitness cost have been considered an efficient adaptation strategy of fungi in human host or in the environment under long-term drug treatment conditions [48,49].

In 30thK2 and 26thV1, overexpression of *cdr4* might play a vital role in azole resistance as the *cdr4* deletion strains from 30thK2 and 26thV1 showed a hypersensitive phenotype (Figure 6A), which is similar to the *cdr4* deletion strain from WT. In addition, sequence alterations in the genes, including *NCU02548*, *NCU03491,* and *NCU02051*, may be involved in azole or Terb resistance in 30thK2 and 26thV1. *NCU02548* and *NCU03491* are predicted to be involved in rRNA processing and mRNA processing, respectively (https://fungidb.org/fungidb/app accessed on 24 December 2021), while the functions of these genes and their homologous genes in drug resistance have not been reported. *NCU02051* encodes a hypothetical protein containing 11 transmembrane regions and 2 ATPases associated with a variety of cellular activities (AAA) domains, suggesting the sequence variation of *NCU02051* may be related to transportation for azoles and Terb. Mutations of *NCU01997* and *NCU09308* likely contribute to Terb resistance in 30thK2, while mutations of *NCU01967* and *NCU02867* probably play roles in azole/Terb resistance in 26thV1 (Table 2 and Figure S4). Function prediction suggests a biological process of mitochondrial tRNA threonylcarbamoyladenosine modification (*NCU09308*), and tRNA wobble uridine modification (*NCU01967*) is also likely involved in azole/Terb resistance. The mutations found in this work likely function during the adaptive evolution to the azoles, which may provide a better understanding of drug resistance in pathogenic filamentous fungi.

Although server defects in growth and sporulation exist, the 24thV2 strain is significantly resistant to all the tested chemicals, especially to KTC and VRC. The markedly increased expression levels of *erg11* and *cdr4* may largely contribute to azole resistance, and overexpression of *erg1* should play a role in Terb resistance in 24thV2. However, the changed morphology in the 24thV2 strain suggests this strain contains complicated mechanisms of drug resistance.

It has been considered that the development of drug resistance is the evolutionary dynamic of a population transforming from gene expression “noise”, epigenetic mechanisms, and gene mutation mechanisms—a process that tends towards genetic stability [22,50]. Notably, overexpression of the drug efflux pump gene *cdr4* was necessary for evolutionary adaptation to the azole stress in *N. crassa* as significantly increased transcript levels of *cdr4* were detected in all four evolved resistant strains. Azoles inhibit ERG11 and thus reduce ergosterol biosynthesis. After KTC treatment for 12 h, all four evolved strains showed less disruption than WT in accumulation of ergosterol and sterol intermediates (Figure 4), indicating the stable fitness states under azole stress in the evolved strains.

For the mechanism of occurrence of PoxB resistance under KTC or VRC stress, we excluded the contribution of the targets of PoxB. Transcript levels of chitin synthesis genes in the four evolved strains did not show higher levels than in WT. Chitin synthetases from the resistant and the sensitive isolates of *Alternaria kikuchiana* share similar affinity with PoxB in vitro [44]. Thus, genetic modification of chitin synthetase genes or their expressional changes might not be general causes of PoxB resistance. Transcriptional analysis and drug susceptibility test suggest that overexpression of *atrf-2*, which promotes drug efflux, may contribute to PoxB resistance in 30thK1, 30thK2, and 26thV1. Considering PoxB resistance might be related to peptide transporters in *C. albicans* [45,46], we conjectured that the changes of transcript levels of genes in the peptide transporter system might be relative to the mechanism of PoxB resistance in *N. crassa*. Notably, the single gene deletion mutants for *msf-9* or *NCU03171*, two peptide transporter-encoding genes, were found to be resistant to PoxB for the first time. The down-regulation of transcripts levels of *msf-9* and *NCU03171* may be a strategy to shut down the channel for PoxB to enter cells. Besides, our results suggest that the mutation in *NCU01967*, which is related to elongator complex protein 6, might play a role in PoxB resistance in 26thV1. Roles of NCU01967 and its homologues in drug resistance have not reported.

The reported MBC-resistant strains are caused by mutations of the target gene or overexpression of an ATP-binding cassette transporter [40,51]. However, no significant expression change and no mutation in gene *β-tubulin* were detected in evolved strains, and deletion of *cdr4* (the homologous gene of *ifT1*) did not change sensitivity to MBC, suggesting other mechanisms of MBC resistance in the evolved strains exist.

AmB binds to ergosterol in the cell membrane, and reduction of ergosterol content is a resistance strategy to the polyenes [52], which is opposite to a common adaptive strategy to azoles. In four evolved resistant strains, the mechanism of AmB resistance was irrelevant to the binding target since the total ergosterol amounts in the four drug-resistant strains did not change compared to WT (Figure 4). It has been reported that increased capability to eliminate ROS contributed to AmB resistance in *Aspergillus terreus* [53]. Among the tested genes encoding peroxidase or catalase, the expression levels of *cat-1*, *cat-3,* and *cat-4* in evolved resistant strains were changed relative to those in WT (Figure S5), suggesting the changes of the redox state in the cells of the evolved strains may contribute to AmB resistance. In addition, sequence variations in *NCU02867* and *NCU02058* might contribute to AmB resistance in 26thV1 and 30thK1, respectively. These two genes and their homologues have not been well characterized. NCU02058 protein was predicted to be involved in regulation of mitotic metaphase/anaphase transition (https://fungidb.org/fungidb/app/record/gene/NCU02058#WolfPsortForm accessed on 24 December 2021).

Our results demonstrate that experimental evolution is a powerful method to acquire resistant strains for exploring the mechanism of drug resistance. All four evolved strains developed multidrug resistance to antifungal drugs in different categories, providing a knowledge base of safe drug application in clinic and agriculture settings. Overexpression of *cdr4* is an important adaptive strategy to the stress of KTC or VRC in the four *N. crassa* multidrug-resistant strains obtained from experimental evolution. Moreover, by the convenience of the next-generation genome resequencing and mutant library of *N. crassa*, 16 potential novel genes contributing to drug resistance are identified in our work. These genes include *NCU02058*, *NCU02867*, *NCU02026*, *NCU02060*, *NCU02051*, *NCU01993*, *NCU01967*, *NCU02055*, *NCU02012*, *NCU03246*, *NCU02548*, *NCU03491*, *NCU02052*, *NCU01947*, *NCU01997,* and *NCU09308* (Figure S4). Although their roles in drug resistance still need further investigation, the data related to these genes in this study will be useful for analyzing drug-resistant isolates in clinic and agricultural fields.

## Figures and Tables

**Table 1:** Strains used in this work.

Strains	Source	Genotype	MIC_KTC_^1^ (μg/mL)	MIC_VRC_^2^ (μg/mL)
Wild type (FGSC#4200)	FGSC (Fungal Genetics Stock Center)	Prototrophic	3.65	1.4
30thC1	this study	Prototrophic	3.65	1.4
30thC2	this study	Prototrophic	3.65	1.4
30thK1	this study	Prototrophic	9	4.8
30thK2	this study	Prototrophic	10.8	4.6
26thV1	this study	Prototrophic	11.8	6.4
24thV2	this study	Prototrophic	20	9.5
*cdr4*^KO^ (FGSC#11238)	FGSC	*cdr4* knockout		
*cdr4* ^OE^	this study	*cdr4* overexpression		
30thK1-*cdr4*^KO^	this study	*cdr4* knockout		
30thK2-*cdr4*^KO^	this study	*cdr4* knockout		
26thV1-*cdr4*^KO^	this study	*cdr4* knockout		

^1^ the MIC values for KTC; ^2^ the MIC values for VRC.

**Table 2:** Summary of mechanisms of multidrug resistance in the four evolved strains.

	Terb Resistance			Azole Resistance			AmB Resistance	PoxB Resistance	
	Target	Transporter	Others	Target	Transporter	Others	Others	Transporter	Others
30thK1	*erg1* (SUR) ^1^	*cdr4* (UR) ^2^	mutations in *NCU02058*, *NCU02052*, *NCU02060*, *NCU02051*, *NCU02052*, *NCU02026*, *NCU01947*	*erg11* (SUR)	*cdr4* (UR)	mutations in *NCU02058, NCU02060, NCU02055, NCU02051, NCU02026, NCU02012, NCU01993 NCU03246*	transcriptional changes in *cat-1/ 3/ 4*; mutations in *NCU02058*	*atrf-2* (UR); DR ^3^: *NCU03171*, *mfs-9*, *mfs-8*, *NCU08397* and *opt-1*	
30thK2		*cdr4* (UR)	mutations in *NCU09308, NCU02051, NCU02026, NCU01997*		*cdr4* (UR)	mutations in *NCU02548, NCU03491, NCU02051, NCU02026, NCU02012, NCU01993*	transcriptional changes in *cat-1/ 3/ 4*	*atrf-2* (UR); DR: *mfs-8*, *opt-1* and *NCU08397*	

26thV1	*cdr4* (UR)		mutations in *NCU02051, NCU02867*	*cdr4* (UR)		mutations in *NCU02548, NCU03491, NCU02051, NCU01967, NCU02867*	mutations in *NCU02548, NCU03491, NCU02051, NCU01967, NCU02867*	*atrf-2* (UR); DR: *mfs-8*, *NCU08397* and *opt-1*	mutations in *NCU01967*
24thV2	*erg1* (SUR)	*cdr4* (UR)		*erg11* (UR)	*cdr4* (UR)		transcriptional change in *cat-1/ 3/ 4*	DR: *NCU03171, mfs-9, mfs-8*, *opt-1* and *NCU08397*	

^1^ SUR: the slightly up-regulated gene; ^2^ UR: the up-regulated gene; ^3^ DR: the down-regulated gene.

## Data Availability

Data are included in the article and Supplementary Materials.

## Supplementary Materials

The following supporting information can be downloaded at: https://www.mdpi.com/article/10.3390/jof8020198/s1, Figure S1: *N. crassa* acquired multidrug resistance under azole stress; Table S1: Primers used for construction and verification of *cdr4* deletion strain; Figure S2: Drug susceptibility test of gene knockout mutants for transmembrane transporters; Table S2: Gene specific primers used for qRT-PCR; Figure S3: Effects of *cdr4* deletion or overexpression on drug susceptibility; Table S3: Transcript levels of genes encoding oligopeptide (OPT) transporters and peptide transporters (PTR/POT); Figure S4: Drug susceptibility test of knockout mutants of genes with SNPs or Indels in the evolved resistant strains; Table S4: Summary of SNPs and Indel mutations in the tested strains; Figure S5: Transcript levels of catalase encoding genes in the evolved resistant strains and WT; Table S5: The information of SNPs; Table S6: The information of the Indels.
