# Supplementary material for: Experimental Evolution of Multidrug Resistance in Neurospora crassa under Antifungal Azole Stress
Source: J Fungi (Basel). 2022 Feb 18;8(2):198. doi: 10.3390/jof8020198 (PMC8875772; doi:10.3390/jof8020198)
Supplement: Supplementary file 1 [file jof-08-00198-s001.zip › jof-1585599-supplementary-final.pdf]

Journal of Fungi

MDPI

Supplementary materials

Contents:

Table S1: Primers used for construction and verification of *cdr4* deletion strain.
Table S2: Gene specific primers used for qRT-PCR.
Table S3: Transcript levels of genes encoding oligopeptide (OPT) transporters and peptide transporters (PTR/POT).
Table S4: Summary of SNPs and Indel mutations in the tested strains.
Table S5: The information of SNPs.
Table S6: The information of the Indels.
Figure S1: *N. crassa* acquired multidrug resistance under azole stress.
Figure S2: Drug susceptibility test of gene knockout mutants for transmembrane transporters.
Figure S3: Effects of *cdr4* deletion or overexpression on drug susceptibility.
Figure S4: Drug susceptibility test of knockout mutants of genes with SNPs or Indels in the evolved resistant strains.
Figure S5: Transcript levels of catalase encoding genes in the evolved resistant strains and WT.

Table S1. Primers used for construction and verification of *cdr4* deletion strains.

Primer name	Sequence(5′→3′)	Note
cdr4^{KO}-5F	GTAACGCCAGGGTTTTCCCAGTCACGACGGGTAACGAG-TATATCCGTGG	For construction of *cdr4* deletion strain
cdr4^{KO}-5R	ATCCACTTAACGTTACTGAAATCTCCAACGACGAGTACAG-TGACGAAGG	
cdr4^{KO}-3F	CTCCTTCAATATCATCTTCTGTCTCCGACGAAGAGAGAGAA-TAGCGAGG	
cdr4^{KO}-3R	GCGGATAACAATTTCACACAGGAAACAGCAGAGACCACAAC-GTAACTGC	
cdr4^{KO}-HphF	GTCGGAGACAGAAGATGATATTGAAGGAGC	
cdr4^{KO}-HphR	GTTGGAGATTTCAGTAACGTTAAGTGGAT	
cdr4^{KO}-Vef F1	CGAAGTCGATACCCATTTCCC	For transformant verification by PCR
cdr4^{KO}-Vef R1	CTTGTATGGAGCAGCAGACGC	
cdr4^{KO}-Vef F2	TTGTGATCCGCCTGGACGACT	
cdr4^{KO}-Vef R2	ACCACCGAACATCCGAAACGA	

Table S2. Gene specific primers used for qRT-PCR.

Gene	Locus No.	Forward primer(5′→ 3′)	Reverse primer(5′→ 3′)	Source
β-tubulin	NCU04540	CCCAA-GAACATGATGGCTGCTTCT	TTGTTCTGAACGTTGCG-CATCTGG	
erg11	NCU02624	AAATCGATTACGGCTAC-GGTCTCG	TATCGCTACCATCCAC-GTTCCTGA	(1)
cdr4	NCU05591	GCTTTGGAAATGGATGGTGAC-GCT	AAATGCAGAGGGCGGTCTTA-GAGT	
erg1	NCU08280	CGTGGTGCTGGCGAGACATTA	CCTCCTTCCAAATCGTCGGCA	
chs-1	NCU03611	GTCGACCTACATCAACATCC	GTGGCTTCTCAATCTCTTCC	
chs-3	NCU04251	CACGGTTGTACATGGGTATG	GAGACGATGAGGGTGTAGAA	
chs-4	NCU09324	CCACACACTCTCGTTTCTC	GCTTGACCGACTCTCATTT	
abc-8	NCU07546	TTCTATCGCTTCTGGATGATTG	GCCAGTGAGTCCTTGTAATG	
abc-3	NCU09975	TCGTCTTTGGCGCTTTAC	TAGCCAGTGACGAGATAGTT	
atrf-2	NCU10009	GGAGTACATGGAAC-CTTTCTTC	CAAATCCCTCCACCTGTTATC	
msf-9	NCU05079	TCCTCTTCCTTCCCATCTAC	GTAGGAACAAGAAGGACGATAG	
NCU03171	NCU03171	GCTGTCGACTCTCGTTATATTC	GAGGTCCAAAGATCTCTCCT	
msf-8	NCU08738	TTCGCCATTGGTGGTATTT	GATCGGTAACAACCGAAGAG	
NCU10763	NCU10763	ACAGTTCCTCAGCTCCTT	CATAGGCGAGCCAACATATC	
opt-4	NCU06352	CCAGCACAGCAGCATTTA	CATGTAGTGGCCTAGTTTGAG	This study
NCU08397	NCU08397	GAGGTTACTCTGAACGTCTTG	CGGGATCTTGACATAATGGG	
opt-3	NCU07894	GTGGTTGAGCTGGATCTATG	CCACTCCACACTCACTCTA	
NCU17261	NCU17261	TTCAACTGGATGACATGGA-TAG	GAAGAAAGGCTGGGATAGTG	
opt-5	NCU17269	CGGTGGTGTTTATGGGATAC	ATGGTCACATTAGGCAAGTC	
opt-1	NCU09773	GCATCTGGTCGTATCTCAAC	GCAGCCGAAACCAAGTAA	
opt-2	NCU04991	GCAAGCCATCCAGATGTT	GAATGTCGCGCCGATTAT	
NCU10381	NCU10381	GGCGGTGGGTATGTATAATG	CGATACTCAGGAAACCTTCAC	
NCU09874	NCU09874	CTAATTGCGCGTTCCAAATC	CTCACCGACAAGGTAGTAGT	
cat-1	NCU08791	TACCACCAACCACCCTAA	GCCTGGTTGGCAGAAATA	
cat-3	NCU00355	CCGTCCTAGCCAGATTCTTA	CCCTTGATAACCTCGTCCT	
cat-4	NCU05169	TTAAGGAGACCGGAGAAGAC	CTTCTCAAGCTCATCCAACTC	
vma-1	NCU01207	CCAACAAAATGGCGCCGAG	CCAACTCATACATAGCAACACC	(2)

1. Chen, X.; Xue, W.; Zhou, J.; Zhang, Z.; Wei, S.; Liu, X.; Sun, X.; Wang, W.; Li, S. De-repression of CSP-1 activates adaptive responses to antifungal azoles. *Scientific Reports* **2016**, *6*, doi:10.1038/srep19447.2. Cusick, K.D.; Fitzgerald, L.A.; Pirlo, R.K.; Cockrell, A.L.; Petersen, E.R.; Biffinger, J.C. Selection and Evaluation of Reference Genes for Expression Studies with Quantitative PCR in the Model Fungus Neurospora crassa under Different Environmental Conditions in Continuous Culture. *Plos One* **2014**, *9*, doi:10.1371/journal.pone.0112706.

Table S3. Transcript levels of genes encoding oligopeptide (OPT) transporters and peptide transporters (PTR/POT).

Locus No.	Gene	Function	Homologue encoding gene in *C. albicans*	FPKM_WT	FPKM_WT_PoxB	WT	30thK1	30thK2	26thV1	24thV2
NCU08738	*mfs-8*	MFS peptide transporter	PTR2, PTR22	1.0000	0.414889	1.0000	0.6528	0.5043	0.4861	0.2293
NCU05079	*mfs-9*	MFS peptide transporter	PTR2, PTR22	1.0000	9.347394	1.0000	0.2954	1.3339	1.7093	0.1345
NCU03171		Sexual differentiation process protein isp4	OPT1, OPT4, OPT5, OPT6	1.0000	0.585464	0.9957	0.6238	0.8258	0.8381	0.3725
NCU10763		small oligopeptide transporter	OPT1, OPT4, OPT5, OPT6	1.0000	0.771557	1.0000	0.6651	0.9748	1.0412	1.3482
NCU06352	*opt-4*	OPT-domain-containing protein	OPT1, OPT5	1.0000	0.825135	1.0976	1.0072	1.2861	1.3067	2.3329
NCU08397		unnamed protein product	OPT1, OPT4, OPT5, OPT6	1.0000	0.260375	1.0000	0.0822	0.2007	0.4778	0.0112
NCU07894	*opt-3*	oligopeptide transporter 2	OPT1, OPT4, OPT5, OPT6	1.0000	64.11765	1.0000	0.9208	0.6652	0.6222	1.9504
NCU17261		hypothetical protein	OPT2, OPT3, OPT4, OPT5, OPT6	1.0000	1.450505	1.0000	2.0439	1.4442	2.0969	1.4521
NCU17269	*opt-5*	oligopeptide transporter OPT	OPT5, OPT7	1.0000	0.5	1.0000	0.2397	1.4182	2.5003	1.2930
NCU09773	*opt-1*	oligopeptide transporter-1	OPT5	1.0000	0.053328	1.0000	0.0359	0.0847	0.2659	0.0023
NCU04991	*opt-2*	oligopeptide transporter-2	OPT5	1.0000	0.281222	1.0000	0.9219	0.5394	0.9100	1.4717
NCU10381		oligonucleotide transporter	OPT8	1.0000	0.664119	1.0000	1.1865	1.8073	1.5962	2.2886
NCU09874		hypothetical protein	OPT8	1.0000	1.256375	1.0000	0.9444	1.2600	0.8231	0.1457

Table S4. Summary of SNPs and Indel mutations in the tested strains.

Strain	Synonymous	Nonsynonymous	Insertion	Deletion
WT	256	135	24	40
30thC1	669	318	25	41
26thV1	675	318	25	41
30thK1	673	328	27	42
30thK2	659	318	20	36

Table S5. The information of SNPs.

Position	Ref	Wt	30thc1	26thv1	30thk1	30thk2	Ref_Base<->Sample_Base	Codon_Mutate	Aa_Mutate	Gene_Id	Function	Mutated Strain	Ktc	Terb	Amb	Mbc	Poxb
1510741	G	G	G	G	A	G	G<->A	GGC<->GAC	G<->D	NCU02058	hp	30thK1	R[a]	R	N[c]	N	N
1510776	A	A	A	A	G	A	A<->G	ACG<->GCG	T<->A	NCU02058	hp	30thK1	R	R	N	N	N
3460698	A	A	A	A	G	A	T<->C	CTA<->CCA	L<->P	NCU04410	tRNA-processing-10	30thK1					
1488846	T	T	T	T	G	G	T<->G	AAT<->CAT	N<->H	NCU02065	DUF726 domain-containing protein	30thK1, 30thK2	N	N	N	N	N
1613965	A	A	A	G	G	A	A<->G	ATC<->GTC	I<->V	NCU02034	RIP defective	26thV1, 30thK1	N	N	N	N	N
1604943	A	A	A	G	A	G	T<->C	CTC<->CCC	L<->P	NCU02036	tRNA-splicing endonuclease	26thV1, 30thK2	N	N	S[b]	S	N
2693080	A	A	A	G	A	G	T<->C	TCG<->CCG	S<->P	NCU02548	hp	26thV1, 30thK2	R	N	S	N	N
499289	G	G	G	T	G	T	G<->T	GTA<->TTA	V<->L	NCU03491	RNA splicing factor Pad-1	26thV1, 30thK2	R	N	N	N	N
3037566	G	G	G	A	G	A	C<->T	ACG<->ATG	T<->M	NCU16667	hp	26thV1, 30thK2					
1921495	T	T	T	A	T	A	T<->A	AGT<->AGA	S<->R	NCU03641	Beta-glucosidase 2	26thV1, 30thK2	N	N	N	N	N
1399197	G	G	G	C	G	G	C<->G	CTT<->GTT	L<->V	NCU05591	ABC transporter CDR4	26thV1	S	S	N	N	R

[a] R: The gene knockout mutant showed resistant phenotype (R) to the indicated antifungal drug compared to the WT strain.[b] S: The gene knockout mutant showed hypersensitive phenotype (S) to the indicated antifungal drug compared to the WT strain.[c] N: The gene knockout mutant showed no difference (N) to the indicated antifungal drug relative to the WT strain.

Table S6. The information of the Indels.

Ref_ID	Position	Type	Base	Strand	Gene_Id	Function	Mutated Strains	KTC	Terb	Amb	MBC	Poxb
CM002236	1467874	D4	TACC	+	NCU16336	3-hydroxyacyl-CoA dehydrogenase	30thK1					
CM002236	1489033	I3	GAT	-	NCU02065	DUF726 domain-containing protein	30thK1, 26thV1	N	N	N	N	N
CM002236	1494042	I2	AC	+	NCU02063	mitochondrial intermediate peptidase	30thK1, 30thK2					
CM002236	1504458	D1	G	-	NCU02060	zinc metallopeptidase	30thK1	R[a]	R	N[c]	N	N
CM002236	1504461	D4	GCAC	-	NCU02060	zinc metallopeptidase	30thK1	R	R	N	N	N
CM002236	1523039	D5	GATGA	+	NCU02055	uridine nucleosidase Urh1	30thK1, 26thV1	R	N	N	N	N
CM002236	1538959	D1	T	-	NCU02052	transcription initiation factor TFIId 127kD subunit, variant	30thK1	N	R	N	N	N
CM002236	1543660	D1	T	+	NCU02051	hp	30thK1, 30thK2, 26thV1	R	R	N	N	S[b]
CM002236	1574451	D5	TAGAA	-	NCU02044	GTP-binding protein	30thK1					
CM002236	1580469	D4	GATT	-	NCU02042	sterol-4alpha-carboxylate 3-dehydrogenase (decarboxylating)	30thK1	N	N	N	N	N
CM002236	1580475	I4	CAAC	-	NCU02042	sterol-4alpha-carboxylate 4-dehydrogenase (decarboxylating)	30thK1	N	N	N	N	N
CM002236	1615222	D2	AA	-	NCU02033	hp	30thK1, 26thV1	S	N	S	N	N
CM002236	1637568	D4	CAGG	+	NCU02026	hp	30thK1	R	R	N	N	N
CM002236	1637669	D1	A	+	NCU02026	hp	30thK1, 30thK2	R	R	N	N	N
CM002236	1641288	D9	AACAACAAA	+	NCU02024	hp	30thK1, 30thK2, 26thV1	N	N	N	N	N
CM002236	1641833	I4	AAAC	+	NCU02024	hp	30thK1, 30thK2	N	N	N	N	N
CM002236	1641834	I1	A	+	NCU02024	hp	30thK1	N	N	N	N	N
CM002236	1673214	D1	C	+	NCU02014	hp	30thK1, 26thV1					
CM002236	1673216	D8	TTAAGGAG	+	NCU02014	hp	30thK1, 26thV1					

Ref_ID	Position	Type	Base	Strand	Gene_Id	Function	Mutated Strains	KTC	Terb	Amb	MBC	Poxb
CM002236	1675626	I2	TT	-	NCU02012	hp	30thK1, 30thK2	R	N	S	S	S
CM002236	1704179	I4	GATG	-	NCU02005	phosphoadenosine phosphosulfate reductase	30thK1, 26thV1					
CM002236	1736108	I5	GGCCG	-	NCU14007	hp	30thK1, 26thV1					
CM002236	1749270	D1	A	-	NCU01993	ethanolaminephosphotransferase	30thK1, 30thK2	R	N	N	N	N
CM002236	1818708	I2	TT	+	NCU01973	SET-8	30thK1	N	N	N	N	N
CM002236	1904395	I2	GC	+	NCU01947	hp	30thK1	N	R	N	N	N
CM002237	2943417	D1	T	+	NCU01620	hp	30thK1	N	N	N	N	N
CM002236	5046154	I1	T	+	NCU03246	tyrosine-protein phosphatase CDC14	30thK1	R	N	N	N	S
CM002236	6108158	I1	T	+	NCU07390	hp	30thK1					
CM002236	117982	D4	CTTC	+	NCU08055	glycoside hydrolase family 3 protein ZIP-1	30thK2	N	N	N	N	N
CM002236	1730716	I2	AA	-	NCU01997	ABC transporter	30thK2	N	R	N	N	N
CM002236	1671820	I3	TCC	+	NCU02014	hp	30thK2, 26thV1					
CM002242	3212857	I1	T	-	NCU02243	hp	30thK2, 26thV1	N	N	N	N	N
CM002236	4307175	I1	G	-	NCU09308	glycoprotease	30thK2	N	R	N	N	N
CM002240	5238569	I1	A	-	NCU04216	hp	30thK2, 26thV1					
CM002236	1835539	D1	T	+	NCU01967	hp	26thV1	R	N	S	N	R
CM002236	8866762	I1	G	-	NCU02867	hp	26thV1	R	R	R	N	N
KC683708	14197	D1	C	+	NCU16302	mitochondrial ribosomal protein S5 (mitochondrion)	26thV1					

[a] R: The gene knockout mutant showed resistant phenotype (R) to the indicated antifungal drug compared to the WT strain.[b] S: The gene knockout mutant showed hypersensitive phenotype (S) to the indicated antifungal drug compared to the WT strain.[c] N: The gene knockout mutant showed no difference (N) to the indicated antifungal drug relative to the WT strain.

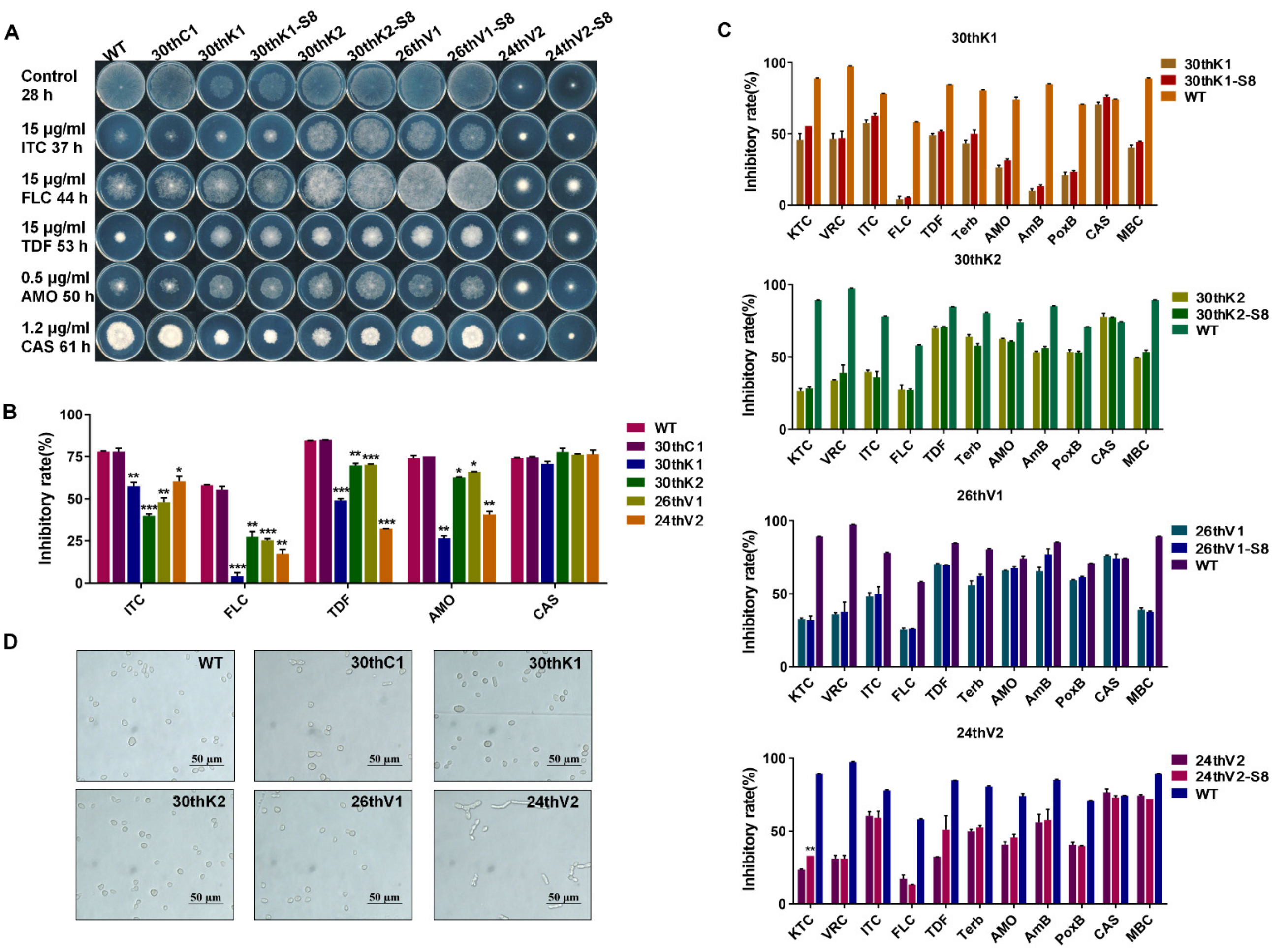

Figure S1. *N. crassa* acquired multidrug resistance under azole stress. (A) Drug susceptibility test of the indicated strains to different antifungals at designated concentrations. Two microliter aliquots of conidial suspension (2×10^6 conidia/mL) were inoculated in the center of plates (ϕ90-mm) with or without the antifungals. The plates were then incubated at 28°C for the indicated time. The experiment was independently repeated at least three times. (B) Relative growth inhibition rates were calculated based on colony diameters at indicated hours after drug treatment. Values from three replicates were used for statistical analysis. Means of the inhibition rates are shown, and standard deviations are marked with error bars. Difference significance between the evolved strains and the ancestral WT strain were estimated by the t-test and marked as * ($p < 0.05$), ** ($p < 0.01$), and *** ($p < 0.001$). (C) Relative growth inhibition rates were calculated based on colony diameters of colonies at indicated growth time. Values from three replicates were used for statistical analysis. Means of the inhibition rates are shown, and standard deviations are marked with error bars. Significance between the evolved strains and the ancestral WT strain was estimated by the t-test. Values with $p < 0.001$, $0.001 < p < 0.01$, and $0.01 < p < 0.05$ are marked with***, ** and *, respectively. (D) The spore morphology of the evolved populations and the ancestral WT strain. The abbreviation the antifungal drugs are explained: ITC (itraconazole), FLC (fluconazole), TDF (triadimefon), AMO (amorolfine), and CAS (caspofungin).

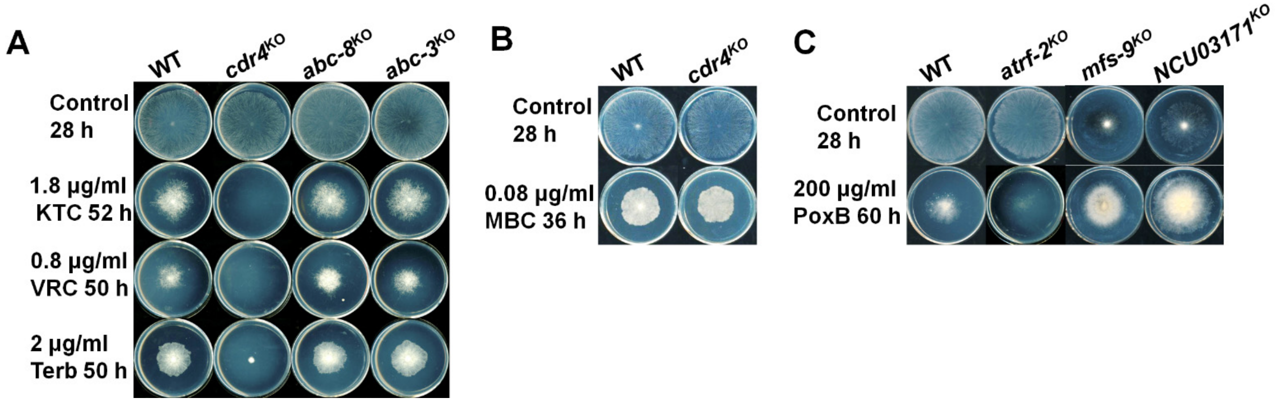

Figure S2. Drug susceptibility test of gene knockout mutants for transmembrane transporters, including (A) *cdr4*^{KO}, *abc-8*^{KO}, *abc-3*^{KO} to KTC, VRC and Terb, (B) *cdr4*^{KO} to MBC, and (C) *atrf-2*^{KO}, *mfs-9*^{KO}, and *NCU03171*^{KO} to PoxB. Two microliter aliquots of conidial suspension (2×10^6 conidia/mL) were inoculated in the center of plates (ϕ90-mm) with or without the antifungals. The plates were then incubated at 28°C for the indicated time. The experiment was independently repeated at least three times.

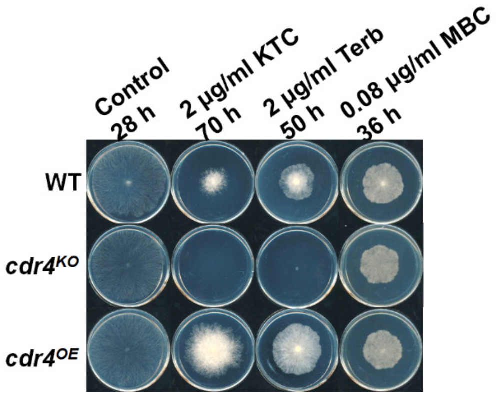

Figure S3. Effects of *cdr4* deletion or overexpression on drug susceptibility. Two microliter aliquots of conidial suspension (2×10^6 conidia/mL) were inoculated in the center of plates (ϕ90-mm) with or without the antifungals. The plates were then incubated at 28°C for the indicated time. The experiment was independently repeated at least three times.

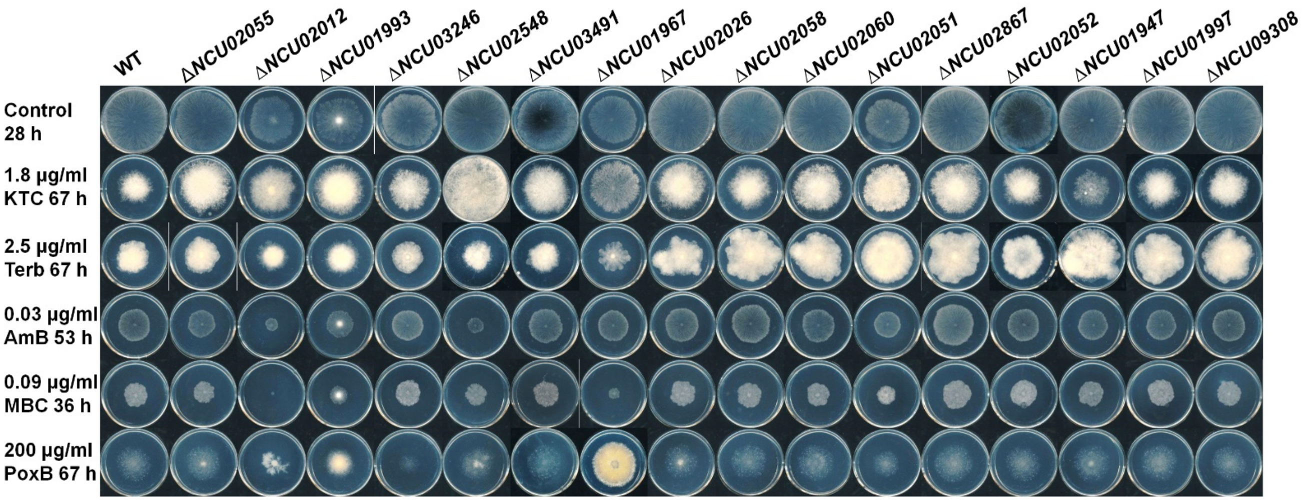

Figure S4. Drug susceptibility test of knockout mutants of genes with SNPs or Indels in the evolved resistant strains. Two microliter aliquots of conidial suspension (2 × 10^6 conidia/mL) were inoculated in the center of plates (ϕ90-mm) with or without the antifungals. The plates were then incubated at 28°C for the indicated time. The experiment was independently repeated at least twice.

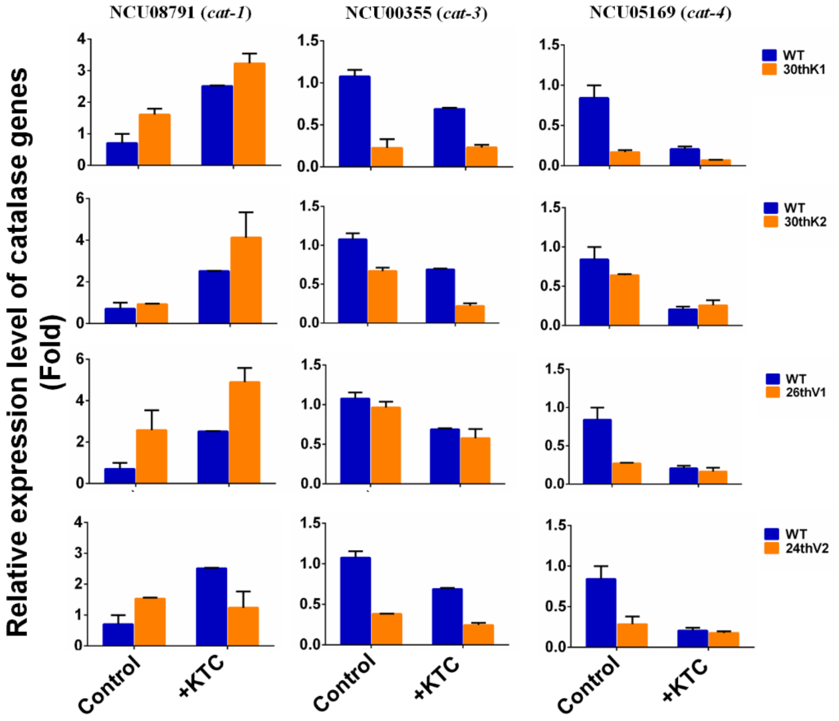

Figure S5. Transcript levels of catalase encoding genes (*cat-1*, *cat-3* and *cat-4*) in the evolved resistant strains and WT. Transcript levels were measured by qRT-PCR, calculated by $2^{-\Delta\Delta Ct}$ method and normalized to *β-tubulin.* The results presented here are means of two biological replicates.
